# From individuals to networks: The role of variation in plant–pollinator communities' responses to global change

**DOI:** 10.1111/1365-2656.70300

**Published:** 2026-06-22

**Authors:** James DeWitt Crall, Marilia Palumbo Gaiarsa

**Affiliations:** ^1^ Department of Entomology College of Agricultural and Life Sciences, University of Wisconsin‐Madison Madison Wisconsin USA; ^2^ Department of Life and Environmental Sciences School of Natural Sciences, University of California, Merced Merced California USA

**Keywords:** biodiversity–ecosystem function, ecological networks, ecological resilience, global change, individual variation, intraspecific variation, plant–pollinator interactions, robustness

## Abstract

1. Plant–pollinator communities are critical for biodiversity, ecosystem function and human well‐being. Yet our ability to predict divergent species responses to environmental change, the risk of abrupt collapse, or the potential for recovery in plant–pollinator systems remains limited.
2. Here, we argue that individual variation within species may play a critical but underappreciated role in shaping the sensitivity, robustness and resilience of animal pollinators and plant–pollinator communities.
3. We explore processes by which individual variation may influence responses to perturbation, highlighting parallels with existing niche theory at the species level (e.g. the biodiversity–ecosystem function literature). We suggest that individual variation—as a key but distinct component of total intraspecific variation—may generate more gradual (rather than abrupt) responses to environmental stress than predictions based on species means in many cases, but that these effects will depend on how traits underlying performance, interaction frequency, stress sensitivity and pollination efficacy covary among individuals.
4. Finally, we highlight critical knowledge gaps and open questions in the structure and dynamics of plant–pollinator interactions at the individual scale and conclude by outlining a roadmap for integrating individual variation into studies of plant–pollinator communities under global change.

## Graphical abstract

This paper argues that variation among individuals—not just species differences—can shape the sensitivity, robustness and resilience of plant–pollinator communities under global change. By linking individual traits and interaction structure to network dynamics, it provides a new framework and future research directions for predicting community responses.

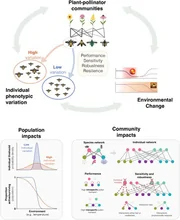

## Resumo

1. Comunidades de plantas e polinizadores são fundamentais para a biodiversidade, o funcionamento dos ecossistemas e o bem‐estar humano. No entanto, nossa capacidade de prever respostas divergentes das espécies às mudanças ambientais, o risco de colapsos abruptos ou o potencial de recuperação em sistemas planta–polinizador ainda é limitada.
2. Neste trabalho, argumentamos que a variação individual dentro das espécies desempenha um papel crítico, embora ainda subestimado, na determinação da sensibilidade, robustez e resiliência tanto dos polinizadores quanto das comunidades planta–polinizador.
3. Exploramos os processos pelos quais a variação individual pode influenciar as respostas a perturbações, destacando paralelos com a teoria de nicho já estabelecida em nível de espécie (por exemplo, a literatura sobre biodiversidade e funcionamento dos ecossistemas—biodiversity–ecosystem function). Sugerimos que a variação individual—como um componente‐chave, mas distinto, da variação intraespecífica total—pode gerar respostas mais graduais (em vez de abruptas) ao estresse ambiental do que aquelas previstas com base nas médias das espécies. No entanto, esses efeitos dependem de como os traços que determinam desempenho, frequência de interação, sensibilidade ao estresse e eficácia da polinização covariam entre os indivíduos.
4. Por fim, destacamos lacunas críticas de conhecimento e questões em aberto sobre a estrutura e a dinâmica das interações planta–polinizador em escala individual e concluímos delineando um roteiro sobre como integrar a variação individual aos estudos de comunidades planta–polinizador sob cenários de mudança global.

## 1 INTRODUCTION

‘*No one supposes that all the individuals of the same species are cast in the same actual mould. These individual differences are of the highest importance for us, for they are often inherited, as must be familiar to every one; and they thus afford materials for natural selection to act on and accumulate’*.

Darwin (1859)

Plant–pollinator interactions underpin the maintenance of terrestrial biodiversity and ecosystem function. Pollination mediates reproduction in most flowering plants and supports the stability and quality of yields for many crops important to human diets and livelihoods (Klein et al., 2007; Ollerton et al., 2011). Predicting if or when plant–pollinator communities might collapse in response to rapidly changing environmental conditions is crucial for conserving biodiversity and supporting human well‐being (Potts et al., 2010; Timberlake et al., 2026). Despite the emergence of plant–pollinator systems as a model for the ecology of mutualistic networks in recent decades, our ability to predict the impacts of rapid environmental change on pollinators—and the consequences for ecological communities or ecosystem services—remains limited (Bascompte & Scheffer, 2023; Peralta, CaraDonna, et al., 2024). Among pollinators, closely related or apparently similar species show markedly divergent responses to environmental change, creating patterns of ‘winners and losers’ (Jackson et al., 2022). Yet what makes some taxa resilient and others susceptible to different extinction drivers remains poorly understood. At the same time, understanding and possibly predicting the risk of collapse in plant–pollinator systems (e.g., ‘tipping points’; Huang et al., 2021), the pace of decline after critical thresholds are exceeded, and the potential for recovery after disturbance are well‐recognized challenges of paramount importance. However, empirical evidence for such critical thresholds remains largely theoretical (Burkle et al., 2013; Lever et al., 2014), and we have only a limited understanding of factors underlying tolerance to environmental change. Understanding the mechanisms underlying sensitivity and resilience to environmental change is key to accurately predicting and effectively mitigating the effects of global environmental change on biodiversity, while also identifying ‘safe operating spaces’ that maintain stable pollination services and human well‐being (Steffen et al., 2015; Timberlake et al., 2026).

Individual variation within species can shape sensitivity to perturbation and capacity for rewiring and recovery (Cantwell‐Jones et al., 2024) and may be critical to addressing these knowledge gaps, yet has received limited attention in the literature on plant–pollinator interactions. More than 150 years ago, Darwin recognized the importance of within‐species variation in driving evolution. This emphasis on variation among individuals marked a fundamental departure from the Linnaean focus on differences between discrete groups (i.e., species), but despite Darwin's early emphasis on variation, modern ecological theory has focused primarily on mean differences between species. Recent years, however, have reignited the emphasis on individual‐level variation, recognizing its ubiquity (Bolnick et al., 2003; Des Roches et al., 2018; Sih et al., 2004)—arising even in the absence of genotypic or environmental variation (Ayroles et al., 2015; de Bivort, 2025)—and impact on ecological and evolutionary processes (Cantwell‐Jones et al., 2024; Gamelon et al., 2025; Gomez & Perfectti, 2012; Violle et al., 2012) from collective performance in social animals (Jolles et al., 2020; Oster & Wilson, 1978) to species interactions (Bolnick et al., 2003; Gaiarsa et al., 2022; Guimarães, 2020). In parallel, the increasing dominance of global environmental change has shown the limits of the classical, static view of ecological systems under equilibrium conditions, shifting the emphasis towards understanding how species and communities respond to—and recover from—perturbations. A key challenge arising from this shift is identifying traits that promote robustness and resilience to environmental change, ranging from gene expression and regulatory networks (Vitousek et al., 2025) to species and ecological communities (Bascompte & Scheffer, 2023; Hernández‐Carrasco et al., 2025; Inouye et al., 2021). Here, we suggest that focusing on individual variation may help identify trait‐based mechanisms that buffer sensitivity or promote resilience to environmental change, while also shedding light on the ecological and evolutionary forces that produce phenotypic variation within species in the first place (Des Roches et al., 2018).

In pollinators, individuals vary substantially within species in key phenotypes including morphology (Couvillon & Dornhaus, 2010), physiology (Pimsler et al., 2020), and behaviour and cognition (Chittka et al., 2003). Phenotypic variation underlies species' ecological niches and shapes plant–pollinator interactions (Junker et al., 2013), such as tongue length driving visitation frequency in bees (Peat et al., 2005) and moths (Haverkamp et al., 2016) via trait matching (Klumpers et al., 2019), and wing shape impacting dispersal in butterflies (Breuker et al., 2007). While few studies have explicitly linked intraspecific variation to ecological resilience in plant–pollinator networks, the existing evidence suggests important potential effects. For example, across bumblebees (*Bombus* spp.) species with higher variation in body size are more likely to have stable or increasing population size under anthropogenic environmental change (Austin & Dunlap, 2019). At the community level, the degree of niche complementarity and functional redundancy among individuals may influence species coexistence and community dynamics across different perturbation scenarios (Hart et al., 2016; Poisot et al., 2015; Tur et al., 2014).

**FIGURE 1:**
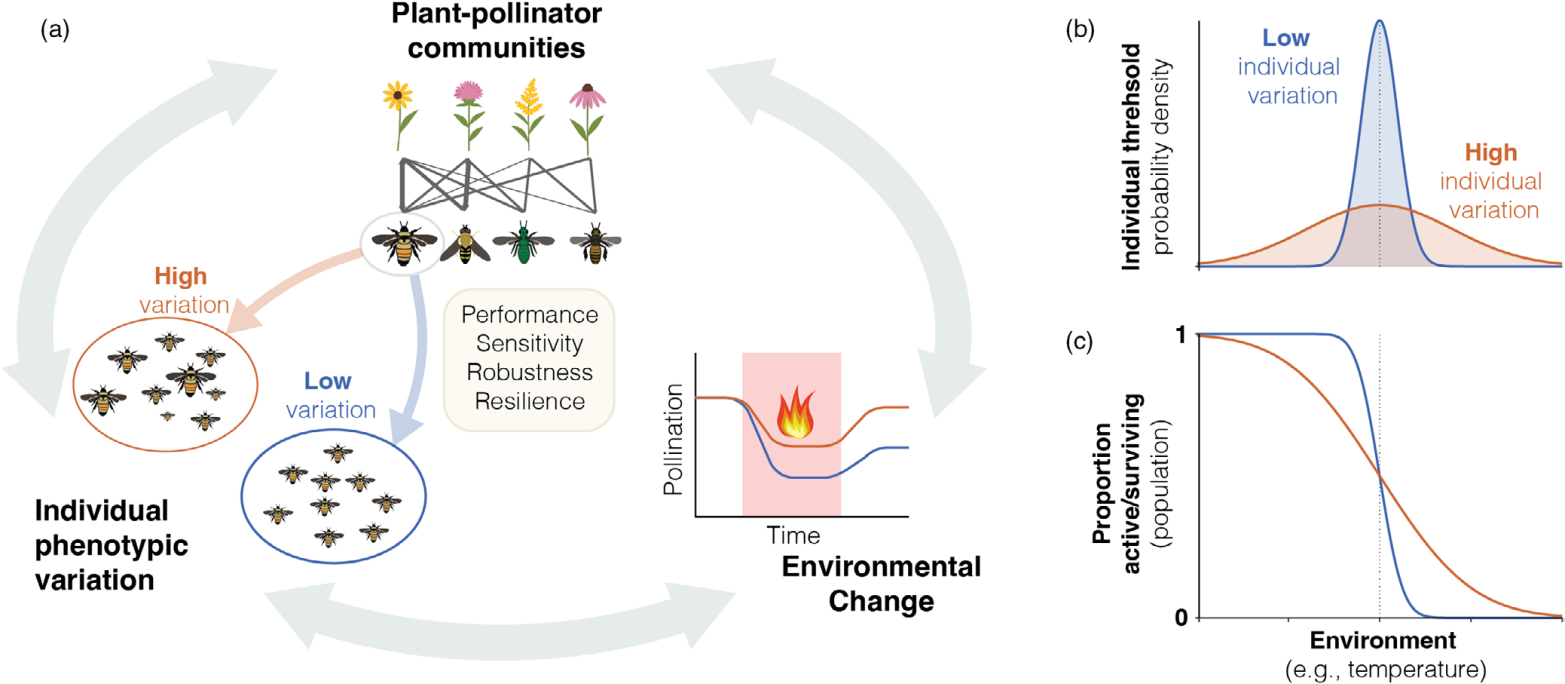
Linking individual phenotypic variation in plant–pollinator communities to environmental change. (a) Individual variation in traits affects the performance, sensitivity, robustness and resilience of plant and pollinator species, ultimately driving population and community responses to environmental change. (b, c) Schematic representation of how trait distributions influence physiological thresholds (e.g. upper thermal limits). (b) Population‐scale sensitivity to environmental conditions (e.g. temperature) may have high (orange) or low (blue) individual trait variation. This, in turn, may result in (c) an abrupt cutoff when the threshold is reached in populations with low individual variation (in blue), and a slow decay in populations with high individual variation, suggesting that these populations can withstand greater environmental variation. While here we focus on pollination and population dynamics, this framework can be applied to other scales and aspects of performance. Graphic art of pollinators by Jeremy Hemberger.

Despite its potential to influence ecological sensitivity and resilience (Figure 1; Cantwell‐Jones et al. 2024; Guimarães, 2020), few studies to date have explored the role of individual variation in shaping the responses of plant–pollinator communities to anthropogenic change, let alone identified underlying mechanisms. Here, we explore the growing empirical evidence for individual variation in plant–pollinator communities (Arroyo‐Correa et al., 2025; Cantwell‐Jones et al., 2024; Gaiarsa et al., 2022; Guimarães, 2020; Peralta, Resasco, et al., 2024) and consequences for ecological sensitivity and resilience. We focus on pollinators because of robust emerging empirical evidence for individual variation, but include plant examples where appropriate to highlight that these patterns and processes are not restricted to pollinators; our intention is to provide illustrative examples of how and why individual variation matters, rather than provide a comprehensive review. We first synthesize perspectives on how individual variation may influence higher‐order characteristics (such as sensitivity, robustness, and resilience) at the population, species, and community scales, drawing parallels with the role of species diversity and niche heterogeneity in ecosystem functioning. We next explore illustrative examples of mechanisms by which individual variation affects the sensitivity, robustness and resilience of plant–pollinator communities under perturbation. Finally, we identify critical open questions and practical future directions that we hope will spur new focus on the role of individual variation in the resilience of plant–pollinator networks. While we focus here on the effect of niche‐based, phenotypic variation on communities' responses to global change as a starting point, plant–pollinator interactions arise from both niche (e.g. trait matching) and neutral processes (e.g. variation in interaction rates driven by population abundance or stochastic fluctuations) (Peralta et al., 2020), and similar dynamics likely occur for neutral processes. We specifically highlight the need to (a) quantify individual variation in empirical systems, (b) identify correlations between phenotypic traits underlying performance at the organismal scale and interaction patterns at the community scale and (c) leverage emerging technologies and theoretical models to address these urgent knowledge gaps.

## 2 A FRAMEWORK FOR LINKING INDIVIDUAL VARIATION TO COMMUNITY DYNAMICS

**FIGURE 2:**
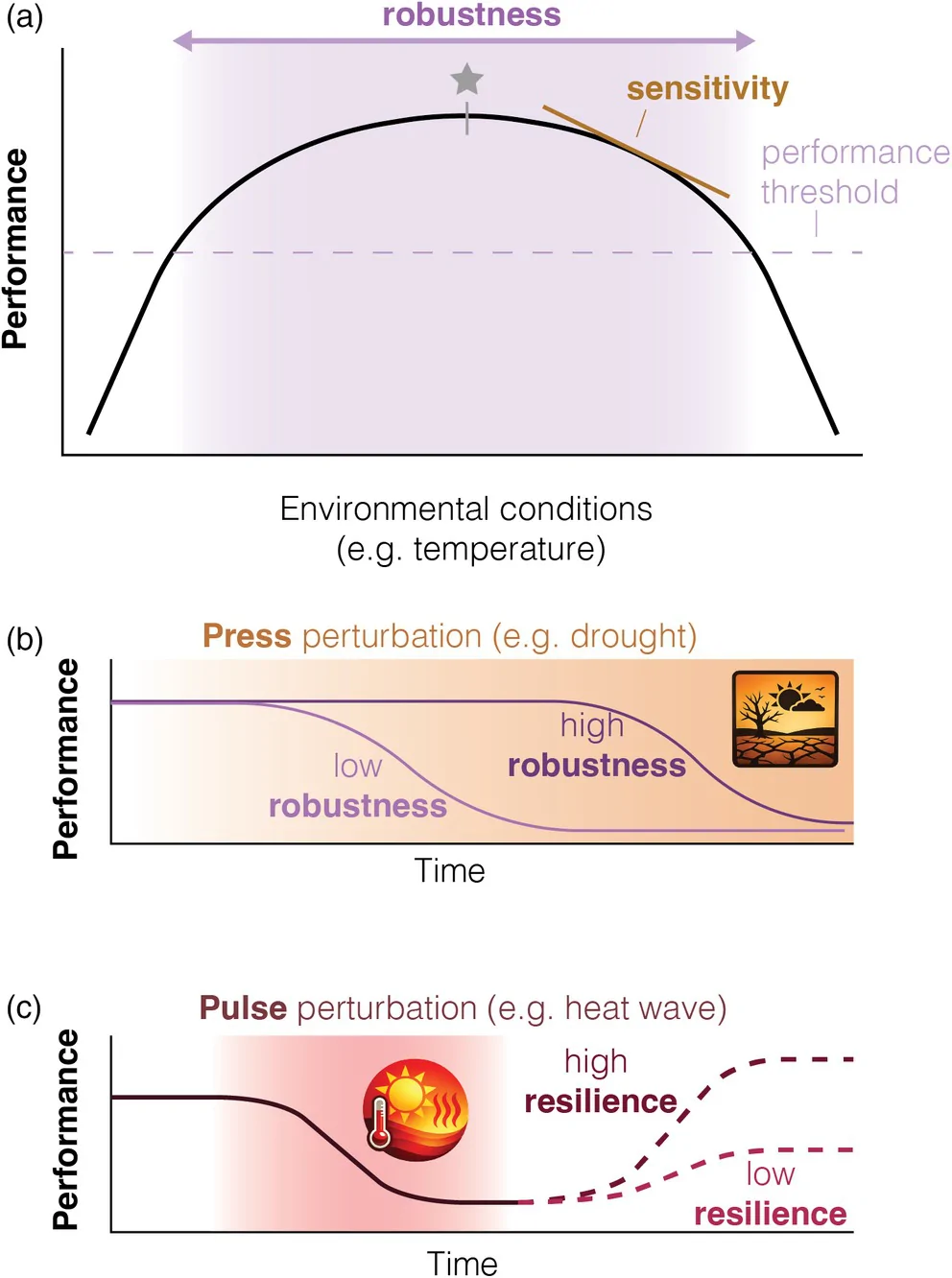
Conceptual framework for key terms. (a) Environmental conditions, such as temperature, can affect pollinator performance, including flight capacity and pollination efficiency. Sensitivity is defined as the rate of change in performance with respect to environmental conditions (i.e. the local slope). Robustness is defined as the range of conditions under which performance remains above a relevant threshold, such as the critical thermal minimum and maximum, with more robust populations exhibiting greater tolerance to a wider range of conditions than less robust populations (b). (c) Resilience is defined as the capacity to recover performance after a perturbation. For example, after the critical thermal maximum is temporarily exceeded, populations with high resilience will return to (or potentially exceed) their pre‐perturbation performance level more quickly, whereas populations with low resilience will take longer—or may never fully recover. Schematic illustrations in (b) and (c) were generated with OpenAI's DALL‐E model.

We focus here on how individual variation affects sensitivity, robustness and resilience at higher levels of biological organization (see Box 1 for operational term definitions used here; Figure 2) (e.g. colonies, populations, species or communities). This framework parallels the biodiversity–ecosystem function (BEF) literature, where well‐established theoretical and empirical work has demonstrated links between species diversity and the productivity and stability of ecological communities. More species‐rich plant communities generally show greater productivity (e.g. biomass production), which may arise through several mechanisms, including niche complementarity (Tilman et al., 2001) or (perhaps more often) sampling effects (diverse communities are more likely to contain highly productive species; Cardinale et al., 2006). Species diversity can also promote stability of ecosystem function (e.g. through biological insurance effects and response diversity; Loreau et al., 2021), akin to widening the stability basin (or ‘robustness’ in our terminology) in Holling's ‘ball‐and‐cup’ analogy (Holling, 1973).

BOX 1 **Definitions of key terms.**

The use of key terms varies across the literature and between fields; here, we adopt the following definitions. We define ***individual variation*** as the component of intraspecific variation attributable to differences between individuals (i.e. ***inter‐individual variation***, equivalent to the between‐individual component of niche variation in Bolnick et al. (2003)). Total intraspecific variation is thus a summation of ***intra‐individual variation*** (e.g. niche breadth within individuals arising from ontogeny, plasticity or other factors) and *inter‐individual variation*. ***Performance*** is the ability of individuals, species or communities to carry out key ecological functions (such as pollination) under given conditions. ***Sensitivity*** describes the degree to which performance shifts in response to changes in the environment (biotic or abiotic) and can be interpreted as the local slope in a performance vs. environment curve. ***Robustness*** refers to the ability of individuals (or communities) to sustain ecological functions under a range of environmental conditions or stress. ***Resilience*** is the capacity to recover functional performance after disturbance, whether through phenotypic plasticity, reorganization (e.g. interaction rewiring) or compensatory dynamics.

Analogous to niche variation across species, individual‐niche complementarity (e.g. the extent to which individuals differ in dietary breadth) and sampling effects (e.g. the probability that a local population contains highly active foragers) may have comparable effects on performance, sensitivity and resilience at the population scale, opening the potential for ‘downscaling’ species niche theory to individuals. However, individual variation may also reflect distinct patterns and processes. For example, physiological or phenotypic trade‐offs may exist only at the level of individuals and not at higher levels (i.e. colony, population or species); while foraging bees exhibit trade‐offs between speed and accuracy in learning that reflect fundamental neurobiological constraints at the individual level (Chittka et al., 2003), colonies of social species benefit from a diversity of cognitive strategies among workers (Burns & Dyer, 2008). As a result, trade‐offs observed at the individual level do not necessarily scale to colonies, populations or species, and vice versa. Thus, diversity‐performance relationships established at one scale (such as performance‐tolerance trade‐offs that support biodiversity‐stability relationships across species; Loreau et al., 2001; Mariotte et al., 2013) will not necessarily translate down to lower scales (e.g. individuals or colonies). It is thus critical to draw on BEF theory while recognizing potential limitations of directly applying species‐level effects to the individual level.

**FIGURE 3:**
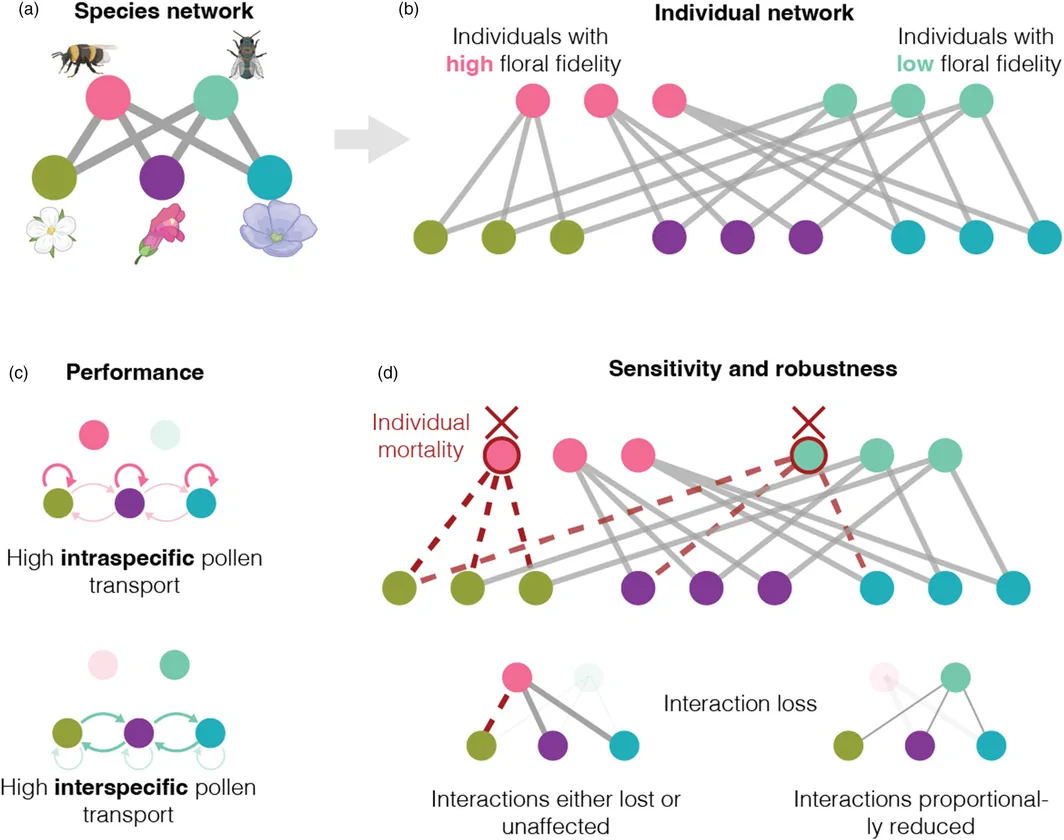
Linking individual variation to species interactions. (a) Representative plant–pollinator community and underlying (b) individual‐level interactions, reflecting two pollinator species composed of ‘specialist’ (high floral fidelity) or ‘generalist’ (low floral fidelity) individuals, and how the resulting (c) pollination service changes based on the predicted pollen transport from different individuals. (d) Individual variation in interaction patterns also affects population responses to the loss of an individual in species‐level interactions. Pollinator and flower illustrations in (a) were generated with BioRender.

To illustrate the importance of variation among individuals in a population—and how it differs from total intraspecific variation—consider a simple plant–pollinator community composed of three plants and two pollinators, which are highly generalist at the species level (i.e. all pollinator species interact with all plant species; Figure 3a). Both pollinator species interact with the same plant species, resulting in identical species‐level interaction patterns. Yet these similar patterns arise from different individual‐level strategies. One pollinator species is composed of individual ‘specialists’, with low overlap in resource use among individuals, leading to high inter‐individual variation, high floral fidelity, and low intra‐individual variation. In contrast, the other pollinator species is composed of individual ‘generalists’, with high overlap in resource use among individuals, leading to low inter‐individual variation, low floral fidelity, and high intra‐individual variation (Figure 3b). These differences in individual‐level interaction structure may lead to substantial differences in pollination services (‘performance’). Highly specialized individuals will repeatedly visit flowers of the same plant species, exhibiting high floral fidelity and thereby leading to greater conspecific pollen transfer relative to generalized individuals (Figure 3c). In this context, perturbations, such as individual mortality, will also have different effects on the two pollinator species. In populations or species composed of individual specialists, interactions could be completely lost, leading to greater sensitivity and potentially lower resilience if dietary preferences are genetically encoded. In contrast, populations composed of individual generalists would show a proportional decrease in interactions until all individuals were lost, reflecting high robustness (potentially at the cost of lower performance; Figure 3d). These patterns would also translate to key aspects of network structure. For example, networks with species containing individual generalists would retain high connectance (the proportion of all potential species links that are filled in a network) until a rapid collapse of connectance at high population loss (Lever et al., 2014). In contrast, in networks with species containing individual specialists, network‐level connectance would be predicted to decline gradually as pollinator populations decline.

## 3 INDIVIDUAL VARIATION AS A DRIVER OF PLANT–POLLINATOR RESPONSES TO GLOBAL CHANGE

Plant–pollinator communities often show divergent responses to similar environmental perturbations. Here, we build on the framework above to explore how individual variation may help explain this heterogeneity in sensitivity, robustness and resilience.

### 3.1 Individual variation and sensitivity

Among both plants and pollinators, closely related and ecologically similar taxa are showing strongly divergent responses to global environmental change. For example, in North America, populations of several bumblebee species are stable or increasing, while others are in decline (Jackson et al., 2022). This pattern appears to be linked to morphological variation within species (Austin & Dunlap, 2019), suggesting that individual phenotypic variation may affect the sensitivity of populations and species to environmental change (Des Roches et al., 2018) through distinct mechanisms.

Generally, when individual variation in stress susceptibility is low (e.g. in thermotolerance), performance at the population scale is expected to show a strongly nonlinear decline near the population‐mean tolerance (Figure 1b,c). In contrast, greater individual variation in susceptibility would yield a consistent decline in performance on either side of the population mean (Figure 1b). As a result, greater individual variation may ‘smooth’ population‐level sensitivity curves, whereas limited variation could promote more threshold‐like responses. Thus, populations with limited individual variation may exhibit a greater tendency towards rapid, threshold‐like responses to environmental perturbations, leading to rapid declines in pollination when critical stress levels are reached. This is consistent with recent work showing that low variation in heat sensitivity within insects (*Drosophila*) leads to more abrupt, threshold‐like declines in survival near the population‐mean tolerance (Bullard et al., 2026).

Individual variation in sensitivity within pollinator species is increasingly well documented; individual pollinators differ markedly in their susceptibility and sensitivity to stressors such as disease (Decanini et al., 2007), temperature (Feuerborn et al., 2023) and pesticide exposure (Easton‐Calabria et al., 2023). Moreover, variation in body size is widespread and can influence sensitivity to stressors, such as starvation (Couvillon & Dornhaus, 2010) and habitat loss (Bommarco et al., 2010). Intraspecific variation in thermal tolerance also varies across populations (Pimsler et al., 2020) and reproductive caste (Feuerborn et al., 2023). While these patterns highlight the ubiquity of intraspecific variation in stress susceptibility, the links between individual‐level variation in susceptibility and tolerance thresholds or sensitivity at the population or species level have not, to our knowledge, been directly tested.

Beyond effects of individual variation on population or species sensitivity, individual variation among pollinators may also influence the sensitivity of communities and species interactions to perturbations (Figure 3). For example, in social and semi‐social bees, division of labour may allow individual foragers to specialize (via high floral fidelity) on particular floral resources (Brosi, 2016; Yourstone et al., 2023), while maintaining dietary diversity at the colony scale within species. In contrast, individual solitary bees must acquire the full range of nutrients for their offspring (Filipiak, 2019). Consequently, they often exhibit low flower fidelity, regardless of resource abundance (Bosch & Vicens, 2005; Torchio & Tepedino, 1980; Williams & Tepedino, 2003). As a result, interactions with individual specialists (e.g. in social species) may yield higher mean pollination efficacy but greater sensitivity (Gomez & Perfectti, 2012; Lopes et al., 2022), echoing productivity–stability trade‐offs in ecosystem services (Montoya et al., 2019).

### 3.2 Individual variation and robustness

**FIGURE 4:**
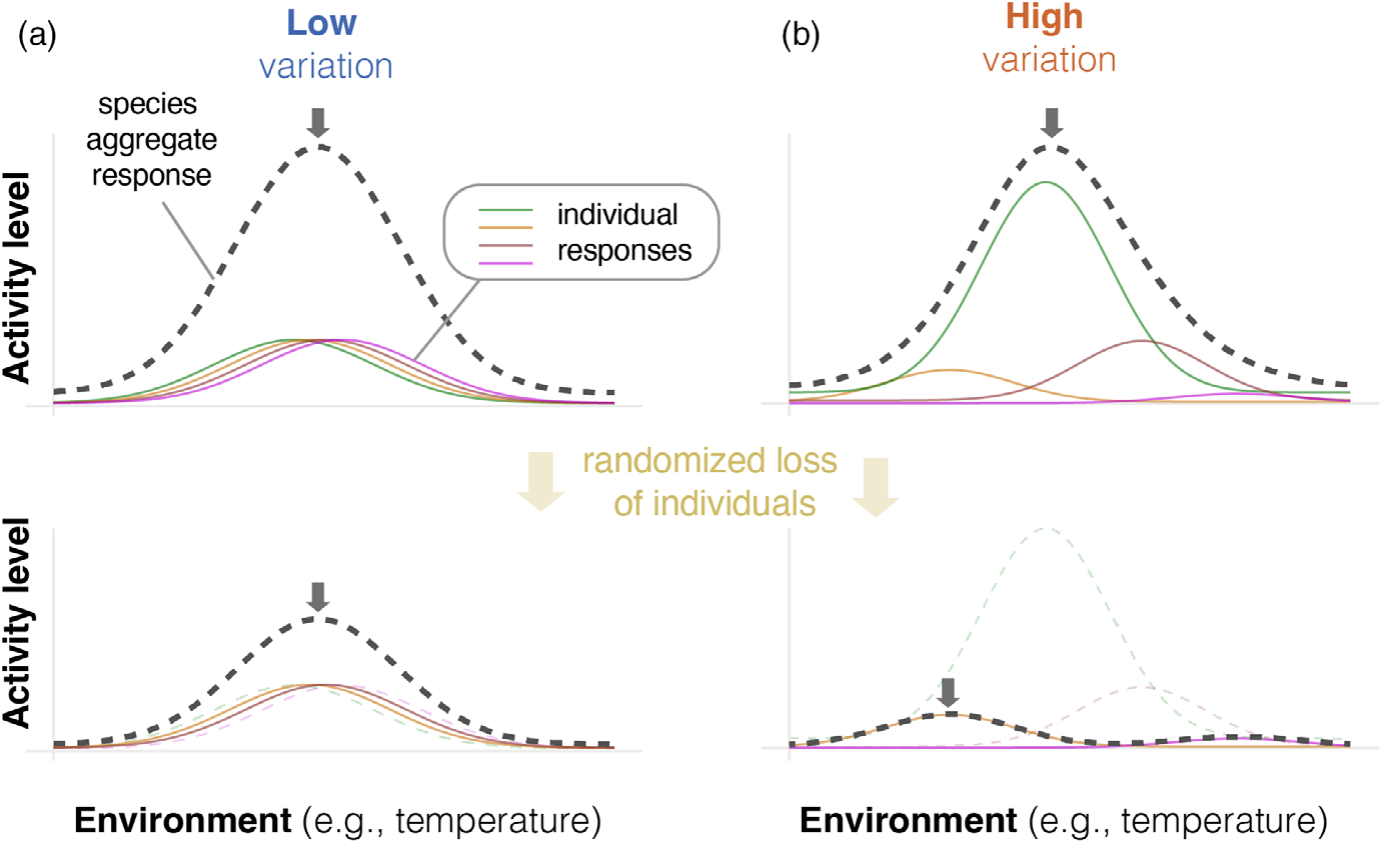
Impacts of individual variation on robustness to the loss of individuals. (a) When individuals have overlapping optima and functional roles (i.e. low individual variation), losses translate into proportional reductions in performance. (b) By contrast, when individuals vary widely in optima or amplitudes, random losses can lead to nonlinear declines and instability in population‐ or community‐level performance. Solid lines show theoretical individual responses, and dashed lines show species aggregate responses. Dark grey arrows indicate peak activity level in each scenario.

Predictions that environmental perturbations could drive the rapid collapse of plant–pollinator communities through cascading loss of interactions and coextinction have become a central theme in pollination ecology (Lever et al., 2014; Memmott et al., 2004). Even though the distribution of individual phenotypes within populations has major implications for robustness and coexistence at the species and community scales (Baruah, 2022; Hart et al., 2016; Figure 4a), empirical evidence for abrupt community‐level collapse or the mechanisms shaping robustness across whole plant–pollinator systems remains surprisingly limited. When individual variation is low, and individuals strongly overlap in traits such as thermal optima or dietary breadth, the loss of individuals can lead to proportional declines in population‐level performance (Figures 1b and 4a). In contrast, when trait distribution is highly variable, even random losses can trigger nonlinear, unstable outcomes in population‐level pollination performance (Figures 1c and 4b). Such effects are conceptually similar to ‘sampling effects’ described at the species scale (Cardinale et al., 2006; Lepš et al., 2006). When individual trait variation is high, performance (e.g. pollination) at the population scale may depend disproportionally on a small subset of highly active individuals, a pattern supported by empirical studies of individual bees (Crall et al., 2018; Tenczar et al., 2014).

However, the extent to which individual loss propagates to the community scale depends not only on how performance varies across individuals, but also on how interactions are organized within networks (Figure 3d). For example, when an individual is removed (Figure 3d), the consequences for community‐level performance depend both on that individual's interaction pattern and complementarity with other individuals. In our hypothetical community shown in Figure 3, individual‐scale network connectance increases after the loss of individuals (0.33 before individual loss vs. 0.38 after; Figure 3b,d, respectively). Superficially, this increase in connectance could suggest greater redundancy in pollination function and increased robustness (Lever et al., 2014). However, explicitly accounting for the interaction patterns of the removed individuals reveals a disproportionate decline in pollination services for some species (e.g. the green plant in Figure 3) not only through reduced visitation but also because the remaining (generalist) pollinators predominantly transport large amounts of heterospecific pollen. Thus, although network‐level metrics may suggest improved system performance, individual‐level contributions to pollination can decline sharply when specialized individuals are lost (Figure 3b).

Generalization at the individual level may buffer interaction loss at the population and species scales (Figure 3d), thereby maintaining temporal patterns in network structure and population‐level performance, and increasing the robustness and persistence of ecological networks (Gaiarsa et al., 2021; Guimarães, 2020; Ponisio et al., 2017). The limited empirical data that exist suggest that indeed intraspecific variation in interaction frequency may promote community robustness: plant population persistence is greater when pollinated by a combination of specialized and generalized individuals (Arroyo‐Correa et al., 2025), and variation in individual phenotypes and in pollinator attraction may increase the feasibility of plant populations (i.e. range of environmental conditions where a community may persist, consistent with ‘robustness’ here; Arroyo‐Correa et al., 2023). A key unresolved question is whether interaction performance covaries with sensitivity: robustness may increase if high‐performance individuals are more stress‐resistant, but decrease if they are disproportionately vulnerable.

Networks of ecological interactions typically exhibit highly skewed distributions, in which most species (and likely most individuals; Bolnick et al., 2003) interact with only a few interaction partners (specialists), while the remainder interact with many partners (generalists). As a result, individual‐level network metrics, such as connectance, nestedness or modularity, may diverge strongly from species‐level averages. While the distribution of interactions has important consequences for network dynamics at the species scale (Bascompte & Jordano, 2007; Gaiarsa & Guimarães, 2019), understanding whether these findings scale down to individual‐level interaction networks can help identify individuals of high functional importance that sustain these critical interactions.

### 3.3 Individual variation and resilience

Plant–pollinator communities show a remarkable capacity to recover following disturbance (Kaiser‐Bunbury et al., 2017), yet these responses are highly variable (Dzekashu et al., 2024), and there is evidence of persistent, long‐term degradation in many systems (Burkle et al., 2013). Individual variation may promote the recovery of populations and species interactions after perturbation through several mechanisms. In the short term, rare behavioural phenotypes (including those that may be suboptimal under normal conditions) and stress‐tolerant individuals can sustain low‐density populations and avert local extinctions through ecological bet‐hedging (Kain et al., 2015). For example, in seed‐dispersal communities, a few plant individuals can attract disproportionately high vertebrate partner diversity, highlighting how rare or specialized individuals can buffer populations against perturbations (Quintero et al., 2025). Individual variation may thus promote (short‐term) resilience by improving population growth at low densities (Chesson, 2000). Over longer timescales (i.e. generations), individual variation may promote evolutionary rescue in response to environmental change and perturbation (Bell, 2013).

High individual variation may also promote population and species persistence and recovery under long‐term, press disturbances (e.g. phenological or range shifts under climate change) by allowing species to fill new (or extirpated) niche space or exploit novel spatio‐temporal overlaps with potential interaction partners by expanding individual variation (Figure 2). For example, species with high trait variance may access a broader set of resources (Peat et al., 2005; Peralta, Resasco, et al., 2024), thereby reducing dependence on any single resource and increasing population‐level resilience (and robustness). Indeed, increased individual‐niche variation was proposed more than half a century ago as a primary mechanism for species‐level niche expansion (Van Valen, 1965). Individual‐niche heterogeneity underlying species‐level niche expansion could provide a plausible mechanism for why some species with higher intraspecific morphological variation exhibit stronger population growth in response to environmental change (Austin & Dunlap, 2019).

## 4 OPEN QUESTIONS

Together, the patterns we outline above suggest that plant–pollinator communities with similar species‐level composition or network structure may nevertheless exhibit dramatic differences in sensitivity, robustness and resilience due to the distribution of phenotypic variation among individuals. Yet many open questions remain, and resolving them will require moving beyond species averages to understand how individual‐level heterogeneity shapes ecological responses to perturbation.

- *What is the empirical distribution of individual trait variation in interactions?* While it is often implicitly assumed that individuals have similar functional efficacy (Gomez & Perfectti, 2012; Herrera, 1987; Lopes et al., 2022; Vázquez et al., 2005), it is likely that individual phenotypic variation (e.g. in behaviour and morphology) may influence pollen deposition (e.g. Figure 3; Section 3.2 above) via differential visitation frequency and interaction efficacy (e.g. pollen deposition). As a result, observed species‐level interaction networks may substantially misestimate functional robustness if interaction frequency and interaction efficacy are unevenly distributed among individuals. For example, communities in which a small subset of individuals disproportionately contribute to pollen deposition may be far more vulnerable to perturbation than species‐level network structure alone would predict. The handful of empirical studies that have directly quantified individual‐level variation in interaction frequency and network position (Arroyo‐Correa et al., 2021; Gaiarsa et al., 2022; Peralta, Resasco, et al., 2024) show strong variation in interaction patterns and functional outcomes for both plants and pollinators. However, empirical studies quantifying individual interactions—especially linking them to other phenotypic traits associated with performance and stress susceptibility—remain quite sparse. Recent technological advances—particularly in computer vision and metabarcoding (see Section 5)—could enable unprecedented resolution to quantify how individual variation in interaction frequency (and potentially efficacy) shapes the dynamics and robustness of ecological communities.
- *How do trait correlations shape performance, sensitivity and resilience?* A second critical gap our framework highlights is how traits underlying performance and interaction frequency correlate with sensitivity at the organismal scale. For example, how does tolerance to environmental stressors (e.g. pesticide exposure) vary between low‐ and high‐performing individuals? In social and semi‐social bees, a small number of individual workers perform the vast majority of foraging bouts (Crall et al., 2018) and floral visits (Thomson & Chittka, 2001). If highly connected or high‐performing individuals are also comparatively stress‐tolerant, ecological function may remain stable despite substantial environmental change. In contrast, if the individuals contributing most strongly to pollination are also the most sensitive to stress, perturbations could generate abrupt declines in pollination despite limited population loss. Quantifying trait correlation structures may reveal trade‐offs between performance and tolerance, analogous to species‐level trade‐offs underlying diversity–stability relationships (Loreau et al., 2021). For example, in plant communities under drought stress, trade‐offs between productivity and drought tolerance across species may promote community stability under fluctuating conditions (Mariotte et al., 2013) (although such trade‐offs are not universal; Jung et al., 2020). However, it remains largely unknown whether similar trade‐offs occur among individuals within populations. Thus, quantifying individual‐level trait covariance is critical and will require integrative studies spanning multiple trait axes—such as disease susceptibility, morphology and cognition. Variation across species in natural history may play an important role in structuring these trade‐offs: for example, sociality in pollinators may affect interaction patterns (see Section 3.1; Figure 3) as well as stress sensitivity (Crall & Raine, 2023). Critically, this will also require moving beyond static morphology to dynamic, multi‐metric measures of performance that link sensitivity‐relevant traits (e.g. disease or thermal susceptibility) to population‐ and community‐level consequences, including interaction structure, functional outcomes and the consistency of organisms' roles in interaction networks (Gaiarsa et al., 2025). These correlations can reveal how selection within‐species cascades to shape the structure and dynamics of mutualistic interactions.
- *How do temporal changes in individual variation shape responses to environmental conditions?* Phenotypic plasticity can rapidly reshape trait distributions across short ecological scales—from hours to seasons. The effect of plasticity will depend on how individual plants and animals differ in their responses (e.g. developmental or behavioural) to environmental change (i.e. ‘reaction norms’; Dingemanse et al., 2010; Cabirol et al., 2023; Suter & Widmer, 2013) and may play a critical role in buffering higher levels of organization (e.g. colonies, populations or communities). For example, within social bee colonies, individual mortality may trigger compensatory upregulation of activity among other workers, providing colony‐level buffering. Individual pollinators can also shift their floral visitation patterns (and degree of ‘specialization’, or floral fidelity) in response to local resource availability and weather (Brosi, 2016; Rose‐Person et al., 2025; Slominski & Burkle, 2021; Spiesman & Gratton, 2016). Thus, the existence of individual variation in behavioural compensation (i.e. ‘behavioural reaction norms’) may buffer pollination function at the population level, even in the absence of colony‐level coordination, functionally equivalent to ‘response diversity’ across species. Alternatively, the loss of individuals may amplify effects at higher levels (e.g. colony or population); in social insect colonies, the loss of specialized workers may drive collective colony collapse (Perry et al., 2015). These examples emphasize how individual variation may drive community‐level compensation and regulation across scales (Vitousek et al., 2025), but also underscore that individuals' plastic behavioural responses across space and time remain poorly understood.

## 5 FUTURE DIRECTIONS

Despite growing recognition that individual variation can shape the sensitivity, robustness and resilience of plant–pollinator communities under global change, key gaps remain in our ability to both measure individual phenotypes and interactions at scale and link those data to predictive theory. We suggest that future work should emphasize two complementary priorities: (1) scalable approaches for characterizing individual variation and (2) tighter integration of theory, data and experimentation.

### 5.1 Scale up measurements of individuals in the laboratory and field

A persistent bottleneck lies in the difficulty of repeatedly monitoring large numbers of individuals under ecologically relevant conditions. Emerging tools now facilitate the quantification of individual behaviour and interaction patterns at unprecedented scales. In particular, the convergence of low‐cost camera systems, robust image and video analysis, and deep‐learning algorithms for behavioural quantification could enable high‐throughput monitoring of individual‐scale interactions and activity (e.g. Arroyo‐Correa et al., 2021; Sittinger et al., 2024; Smith et al., 2026; Spiesman et al., 2021). Integrating deep‐learning‐based detection with classical computer vision can further support automated tracking of floral preferences and visitation dynamics for uniquely identified individuals (e.g. Jain et al., 2025; Ulrich et al., 2024), enabling repeated‐measures estimates of specialization, foraging activity, interaction turnover and behavioural plasticity.

In addition, molecular tools such as DNA metabarcoding can complement visual observations by resolving resource use and cryptic interactions, revealing individual‐level dietary patterns and interaction networks (e.g. Argueta‐Guzmán et al., 2026; Gaiarsa et al., 2022). These scalable approaches should support—not replace—the need for natural history and data collection on basic information on trait distributions, phenology and trait covariances, which remain missing for most plant and pollinator species. A promising direction would be to leverage image data from field collections and citizen‐science repositories to quantify phenotypic variation within and across populations and species (e.g. Ostwald et al., 2025).

### 5.2 Integrate theory and data through prediction‐focused synthesis and experimentation

Although theoretical models incorporating individual variation have advanced substantially (e.g. Arroyo‐Correa et al., 2025; Baruah, 2022), they remain constrained by limited empirical estimates of trait distributions, trait covariances and the structure of individual‐level interactions. A critical next step is to bridge species‐scale frameworks—such as BEF and ecological network theory—to the individual level. Central concepts at the species‐level—complementarity, response diversity and sampling effects—have clear analogues at the individual level, but their consequences for community function and dynamics may differ when variation is structured within species rather than among species (e.g. Figure 3). For example, two plant–pollinator communities with identical species richness and similar connectance may differ dramatically in robustness if interaction frequency and pollination efficacy are broadly distributed across individuals in one community but concentrated among a few highly connected or highly effective individuals in another. Understanding when species‐level expectations scale down to the individual level would shed light on how population‐level trait variation affects network stability, persistence and pollination function under environmental change.

Critically, as with the BEF literature, expanding beyond observation to experiments that manipulate the distribution of individual phenotypes within populations could provide causal tests of how environmental perturbations propagate to affect interaction networks and community dynamics. Manipulating the distribution of individual phenotypes within populations may provide an experimentally tractable route to testing collapse thresholds, buffering capacity and resilience mechanisms that are otherwise difficult to study through species‐level diversity manipulations.

In summary, the convergence of scalable monitoring, molecular and image‐based phenotyping, and increasingly mature individual‐based theory creates an opportunity to understand, quantify and ultimately predict the effects of individual variation on plant–pollinator community responses to global change. Integrating these advances can help address current central gaps: Why apparently similar species diverge in their responses to environmental change, what determines tolerance limits and the potential for abrupt declines, and which mechanisms allow populations and communities to persist or recover following disturbance. By linking individual trait distributions to interaction structure and pollination function, the framework we propose here can move us towards a predictive ecology of plant–pollinator communities under global change, advancing fundamental ecological understanding while informing conservation strategies that protect not only species richness but also functional diversity down to the scale of individuals.

## AUTHOR CONTRIBUTIONS

JDC and MPG contributed equally to all aspects of this paper.

## CONFLICT OF INTEREST STATEMENT

The authors have no conflict of interest to declare.

## Data Availability

No data were used in the article.
